# Sarcosine as a Potential Prostate Cancer Biomarker—A Review

**DOI:** 10.3390/ijms140713893

**Published:** 2013-07-04

**Authors:** Natalia Cernei, Zbynek Heger, Jaromir Gumulec, Ondrej Zitka, Michal Masarik, Petr Babula, Tomas Eckschlager, Marie Stiborova, Rene Kizek, Vojtech Adam

**Affiliations:** 1 Central European Institute of Technology, Brno University of Technology, Technicka 3058/10, CZ-61600 Brno, Czech Republic; E-Mails: cernei.natalia3@gmail.com (N.C.); h12009@vfu.cz (Z.H.); j.gumulec@gmail.com (J.G.); zitkao@seznam.cz (O.Z.); masarik@med.muni.cz (M.M.); petr_babula@email.cz (P.B.); kizek@sci.muni.cz (R.K.); 2 Department of Chemistry and Biochemistry, Faculty of Agronomy, Mendel University in Brno, Zemedelska 1, CZ-61300 Brno, Czech Republic; 3 Department of Pathological Physiology, Faculty of Medicine, Masaryk University, Kamenice 5, CZ-61200 Brno, Czech Republic; 4 Department of Paediatric Haematology and Oncology, 2nd Faculty of Medicine, Charles University, and University Hospital Motol, V Uvalu 84, CZ-15006 Prague 5, Czech Republic; E-Mail: tomas.eckschlager@fnmotol.cz; 5 Department of Biochemistry, Faculty of Science, Charles University, Albertov 2030, CZ-12840 Prague 2, Czech Republic; E-Mail: stiborov@natur.cuni.cz

**Keywords:** cancer of prostate, biomarkers, early diagnostic, prostatic specific antigen, non-invasive markers, urine, amino acids

## Abstract

Prostate cancer (CaP) is the most common type of tumour disease in men. Early diagnosis of cancer of the prostate is very important, because the sooner the cancer is detected, the better it is treated. According to that fact, there is great interest in the finding of new markers including amino acids, proteins or nucleic acids. Prostate specific antigen (PSA) is commonly used and is the most important biomarker of CaP. This marker can only be detected in blood and its sensitivity is approximately 80%. Moreover, early stages cannot be diagnosed using this protein. Currently, there does not exist a test for diagnosis of early stages of prostate cancer. This fact motivates us to find markers sensitive to the early stages of CaP, which are easily detected in body fluids including urine. A potential is therefore attributed to the non-protein amino acid sarcosine, which is generated by glycine-*N*-methyltransferase in its biochemical cycle. In this review, we summarize analytical methods for quantification of sarcosine as a CaP marker. Moreover, pathways of the connection of synthesis of sarcosine and CaP development are discussed.

## 1. Introduction

### 1.1. History

Sarcosine, also known as N-methylglycine with the chemical formula CH_3_NHCH_2_COOH, was firstly isolated and named by German chemist Justus von Liebig in 1847. It is a non-proteinogenic amino acid that occurs as an intermediate product in the synthesis and degradation of amino acid glycine [1]. According to the Web of Knowledge database, there is an increasing incidence of keyword sarcosine as well as an increasing number of citations since 2009. This phenomenon confirms growing interest in sarcosine as a potential marker of a prostate cancer. The first publication describing the relationship between changes in level of sarcosine and progression of a prostate cancer was published in Nature by Sreekumar *et al.* in 2009 [1]. Since then sarcosine has been investigated as a new marker of prostate cancer by Issaq *et al.* [2] and has been identified as a metabolite greatly increasing during progression of a prostate cancer and metastatic process; which can be detected in urine [3].

### 1.2. Background

**Figure 1:**
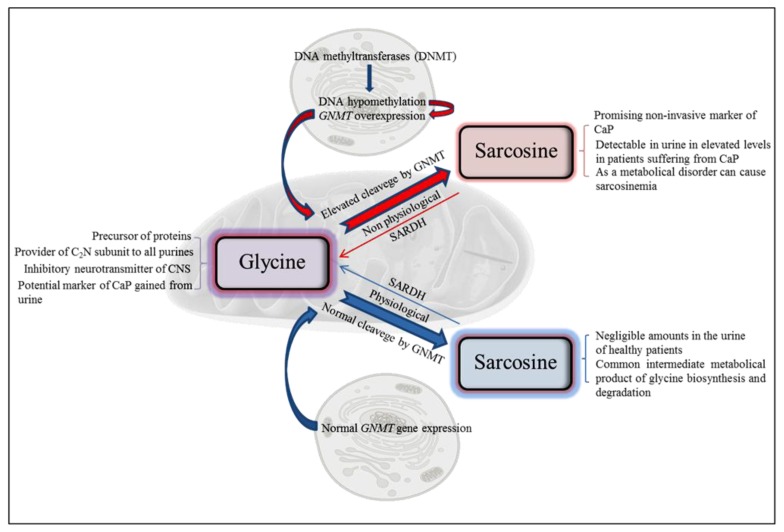
Scheme of biological functions of sarcosine at both physiological and nonphysiological conditions. CaP—prostate carcinoma; GNMT—glycine N-methyltransferase; SARDH—sarcosine dehydrogenase.

According to data describing CaP mortality and incidence in US in year 2013 prostate cancer exhibits the highest incidence (238,590 estimated new cases) and second highest mortality (29,720 estimated deaths) of all cancer types diagnosed in males [4]. Thanks to more developed methods of diagnosis is improved also the prognosis of patients with CaP. Therefore the fact that sarcosine does not occur or occurs in negligible concentration in urine of healthy persons is critical for its evaluation of potential disease marker [1]. This phenomenon reduces the risk of false positive and false negative results [5]. The possibility to use sarcosine as a marker of early stages of development of prostate cancer has been discussed in few papers [6–8]. Thanks to the application of sarcosine as a tumour marker, low cost analytical methods for its determination in the urine, tissue and blood plasma samples are being searched and developed. Biosynthesis of sarcosine has been shown to be certainly affected by the cancerogenesis (Figure 1). This phenomenon was described by Mukherjee *et al.* whose research revealed an important role of glycine N-methyltransferase (GNMT) in the metabolism of tissues of prostate cancer [9]. It has been found that GNMT is involved in the metabolism of methionine as well as in the gluconeogenesis and transformation of sarcosine to glycine (Figure 1). However, the role of sarcosine in carcinogenesis has not been fully understood and remains unknown, as indicate the results of the study published by Hakimi *et al.* [10]. Modulating the function of GNMT can be used to develop new strategies for treatment of prostate cancer. In addition, GNMT could serve as a new tumour marker to diagnose malignant progression of prostate cancer [11]. The most frequently used CaP screening marker—prostate specific antigen (PSA) is organ specific but cannot specify the stage and the type of disease, so its use for prostate cancer screening is insufficient [12]. Therefore, nowadays prostatic antigen 3 (PCA 3) [13–15] and annexin (A3) [5,16–18] are the most widely used and generally accepted markers of non-invasive CaP in urine. There are also other newly discussed potential markers of CaP that may be useful in the diagnosis of progression of prostate cancer, which can be detected in urine, such as alpha-methylacyl-CoA racemase (AMACR) [19–21], which level is elevated in prostatic adenocarcinoma and high-grade intraepithelial neoplasia. TMPRSS2-ETS fusion gene conversions that is very common and specific in alterations in the prostate cancer cells. These genetic alterations lead the overexpression of ETS genes encoding the E26 family of transcription factors involved in cell proliferation [22]. Another used marker is the ratio of free to total PSA—f/tPSA. It was confirmed that this biomarker can better distinguish between patients with prostate cancer from patients with a benign hyperplasia of the prostate [23]. ProPSA—the most stable subform of PSA associated with cancer also exhibits potential, because several studies suggested the clinical usefulness of proPSA in the detection of prostate cancer using different non-commercial assays [24,25]. Some high potential serum biomarkers are: kallikrein 2, urokinase-type plasminogen activator/urokinase-type plasminogen activator receptor, interleukin-6/interleukin-6 receptor, pigment epithelium-derived factor (PEDF), fibronectin 1, chromogranin A ceruloplasmin and others [26,27], nevertheless their clinical utilization and their role in the active surveillance scenario needs to be studied further. The aim of this review is to describe the molecularly-biological aspects and biochemical pathways of biosynthesis of sarcosine and to summarize the basic data about suitability of chosen analytical techniques for determination of sarcosine in various matrixes.

## 2. Methods

**Figure 2:**
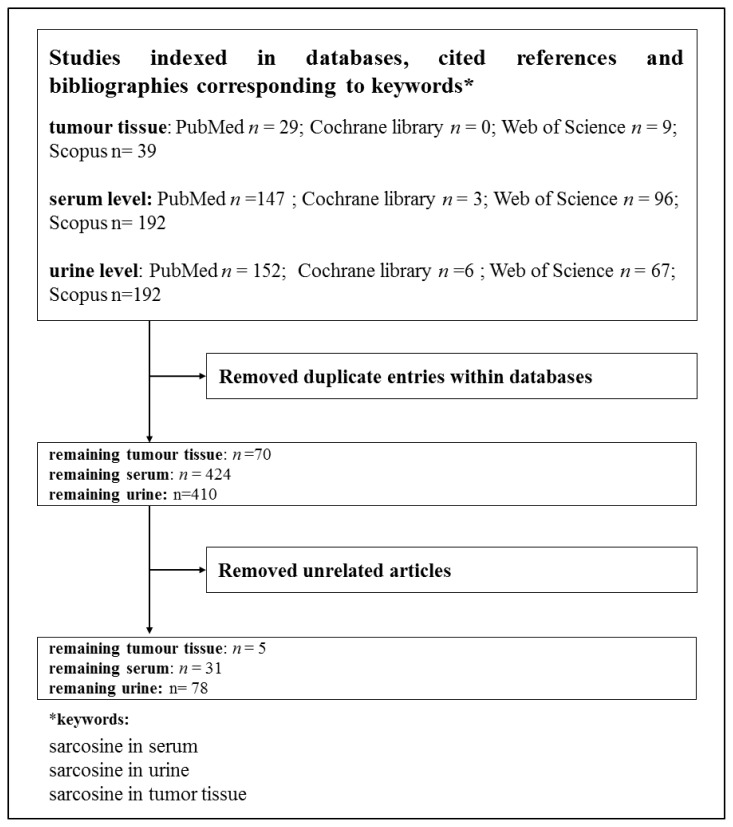
Overview of the databases used for the evaluation of the papers related to the topic of the review.

A literature search covering the topic of the review was performed in following databases: Web of Science (Thomson Reuters, New York, NY, USA), PubMed (United States National Library of Medicine, Bethesda, MD, USA) Cochrane Library (Cochrane Collaboration, Baltimore, MD, USA), and Scopus (Elsevier, Amsterdam, Netherland) as it is shown in Figure 2. The presented figure shows a course of the process of the papers publications devoted to sarcosine. We looked for studies related to sarcosine and its metabolic properties, its molecularly-biological properties and possibilities of its analysis.

The used keywords are shown in Figure 2. Keyword *sarcosine in tumour tissue* gave 77 results in all used databases. Keyword *sarcosine in urine* revealed 417 results in all databases and keyword *sarcosine in serum* provided 438 results within all the databases. Duplicating articles were subtracted for each term from these counts. Seven articles were subtracted for the keyword *sarcosine in tumour tissue*, 14 articles for *sarcosine in serum* and 7 articles for *sarcosine in urine*. The resulting number of articles was subjected to readout of articles that were not directly related to the review topic. We withdrew 65 articles that were not related to the keyword *sarcosine in tumour tissue*, 393 articles for the keyword *sarcosine in serum* and 332 articles for *sarcosine in urine*. The resulting number *n* is the sum of non-recurring articles in all the databases related to the searched keywords. The final numbers were as follows: 5 for *sarcosine in tumour tissue*, 31 for *sarcosine in serum* and 78 for *sarcosine in urine*. These numbers give clear evidence of the importance of sarcosine as a potential biomarker, whose possibility to be determined in urine represents the greatest potential. This fact is important, because urine is one of the most accessible and stable body fluids for analysis [28].

## 3. Molecular Biology of Sarcosine

**Figure 3:**
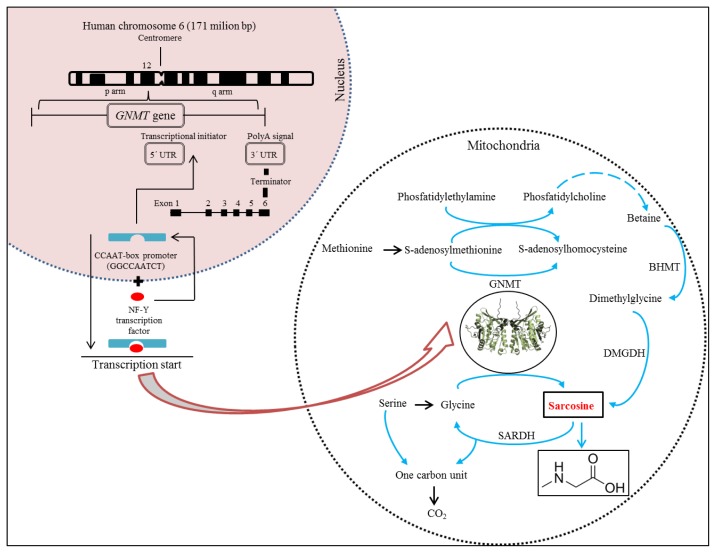
Structure of human cine-N-methyltransferase (GNMT) gene in connection with scheme of sarcosine genesis in biochemical pathway.

Glycine N-methyltransferase (GNMT) acts as an essential component that influences synthesis of sarcosine [29–32]. Synthesis of GNMT is controlled by the same gene named *GNMT*. It has recently been reported that the *GNMT* gene is located on the short (p) arm of chromosome 6 at position 12 (Figure 3) and acts as a tumour-susceptible gene [33]. According to the study by Ianni *et al.*, T allele of the rs9462856 SNP in the promoter region of the *GNMT* gene is overexpressed in patients suffering from CaP and its overexpression significantly increases the risk of the disease [34]. Phenotypic analysis of three *GNMT* haplotypes (A, B, and C) indicated that haplotype C had the highest promoter activity and haplotype B had significantly higher activity compared to the haplotype A. The difference between the haplotypes B and C is due to the T allele of SNP1 that exerts a strong disequilibrium [35]. The *GNMT* gene contains in TATA-less core promoter region the Sp1 site and a CCAAT-box [36] that is one of the most ubiquitous elements being present in 30% of all eukaryotic promoters [37–39]. This region represents a binding site for the transcriptional factor, NF-Y, a trimer with histone-like subunits NF-YB/NF-YC and the sequence-specific NF-YA [40,41]. NF-Y is a sequence-specific transcription factor with nucleosome-like properties in nonspecific DNA binding that helps to establish permissive chromatin modifications at CCAAT promoters [38].

The expression of the *GNMT* induced in this manner leads to synthesis of GNMT that contributes to the regulation of the level of S-adenosylmethionine (SAM) and influences gene expression by affecting the DNA methylation [34]. DNA methylation is an essential process in the body. Methyl groups transferred by SAM are used to synthesize many essential compounds including creatine and/or phosphatidylcholine. In addition, DNA methylation is essential for regulation of gene expression [42]. Deficiency of donors of methyl group (e.g., choline and methionine) or coenzymes of metabolism of methyl group (e.g., folate and vitamin B12) disturbs the intracellular levels of S-adenosylmethionine, triggers the DNA hypomethylation, and promotes cancers of the liver, prostate, and other organs [35,43]. Patients suffering from CaP have been revealed to show a decreased methylation of DNA, whereas patients positive for the *GNMT* T allele had a lower level of methylated DNA than controls with the same allele [34]. GNMT also binds a number of polycyclic aromatic hydrocarbons and inhibits the formation of DNA adducts [30].

Due to properties of GNMT, its excessive production causes a cleavage of glycine to sarcosine and elevated the presence of sarcosine in urine (Figures 1 and 3). Stabler *et al.* reported that increased flux through of GNMT results in the increased formation of homocysteine and sarcosine through increased utilization of SAM [44]. This makes sarcosine interesting in the field of non-invasive cancer biomarkers. Elevated levels of sarcosine correlated with progression of prostate cancer and metastatic process. Supplementation of sarcosine to prostate cancer cell lines induced a selection of invasive phenotype in culture [19]. Dahl *et al.* reported for the first time on a significant upregulation of a potent oncoprotein human epidermal growth factor receptor 2 (HER2/neu) in androgen-dependent prostate cancer cells upon exposure to exogenous sarcosine. That indicates that sarcosine may be involved in regulation of HER2/neu. However, information about these cellular mechanisms will be needed for detailed clarification [45].

## 4. Metabolism of Sarcosine

Metabolites play essential role in an understanding the biological reactions and thereby the changes in their levels contribute to the development of new diagnostic and therapeutic methods to diagnose specific diseases [46,47]. Biochemical pathways of formation and oxidation of sarcosine occur in mitochondria and are provided by two basic pathways (Figure 3). Phosfatidylethylamine is methylated repeatedly by S-adenosylmethionine (SAM) during transulfuration to phosphatidylcholine in the first pathway with the resulting intermediate product betaine. This reaction forms dimethylglycine and regenerates methionine from homocysteine [44]. Dimethylglycine is subsequently converted to sarcosine via dimethylglycine dehydrogenase (DMGDH) [46,48]. The second metabolic pathway creates sarcosine during the transformation of the methyl group of S-adenosylmethionine catalysed by the enzyme glycine-N-methyltransferase (GNMT), that is a tetramer of identical 32 kDa subunits [49,50]. These two reactions ultimately produce 5, 10-methylenetetrahydrofolate and are dependent on oxidized flavoproteins [e.g., flavin adenine dinucleotide (FAD^+^)] [46]. This pathway also includes an oxidative phase, which removes the methyl group from sarcosine to create glycine and an active one-carbon unit by sarcosine dehydrogenase (SARDH), a mitochondrial flavoprotein [51] which, in a study by Montrose *et al.*, was observed within the tumour tissue with GNMT and other enzymes and can explain the increases in sarcosine levels [48]. The absence of the activity of SARDH involves also conversion of choline to glycine in humans; this is transmitted genetically and represents a disorder in metabolism of amino acid that is manifested as sarcosinemia, a surplus of sarcosine accompanied by high concentrations of sarcosine in blood and urine [52].

## 5. Metabolomic of Prostate Cancer

In the last decade, advances in nuclear magnetic resonance spectroscopy (NMR) and mass spectrometry (MS) have been applied to identify metabolomics of prostate cancer that may show at clinically useful biomarkers [53]. NMR exploits the behaviour of molecules when placed in a magnetic field, allowing the identification of different nuclei based on their resonant frequency. MS determines the composition of molecules based on the mass-to-charge ratio in charged particles. The resultant metabolite detection and quantification is acquired as a data set called a spectrum [54]. Tumour metabolomic profile is highly dependent on its organ of origin, and exhibits unique patterns dependent on cancer type as well as differentiation status [55]. Prostate cancer has a distinct metabolic profile characterized by high levels of total choline (tCho) and phosphocholine, together with increasing amounts of the glycolytic products lactate and alanine [56]. In the studyof Swanson *et al.*, healthy glandular tissues demonstrated significantly higher concentrations of citrate and polyamines than healthy stromal and prostate cancer tissues, while healthy glandular and stromal tissues demonstrated lower concentrations of choline, phosphocholine plus glycerophosphocholine and total cholin (tCho) than prostate cancer tissues [57]. The results in cancer metabolomic achieved through *in vivo* MRSI and *ex vivo* NMR investigations during the first 11 years of the 21st century are illustrating the areas where these technologies can be best translated into clinical practice [58].

## 6. Analytical Techniques for Detection of Sarcosine

Currently, new methods for reliable detection of sarcosine have been developed to confirm or contradict sarcosine as a possible marker of prostate cancer [2,7,59].

### 6.1. Microarray-Based Analysis

Microarray technology is based on the assumption that the amount of mRNA for each gene correlates with proteins amount. However, it is well know that a correlation between RNA expression and protein expression rarely holds because of posttranslational modification, protein degradation and feedback of protein on RNA expression [9]. Studying of gene expression provides useful information for both clinical and basic researches [60]. Microarray-based multiplex biomarker analysis of fusion genes in urine samples has been described [5]. Some promising non-invasive biomarkers with potential for detection in the urine were determined as *PCA3*, fusion genes, e.g., *TMPRSS2:ETS*, *TERT*, *AMACR*, *GSTP1*, *MMP9*, *VEGF*, *ANXA3* and sarcosine [5]. The new urine tests should be useful in early diagnosis of prostate cancer and detection of aggressive tumours with the goal of radically improving treatment of CaP.

### 6.2. Chromatography

**Figure 4:**
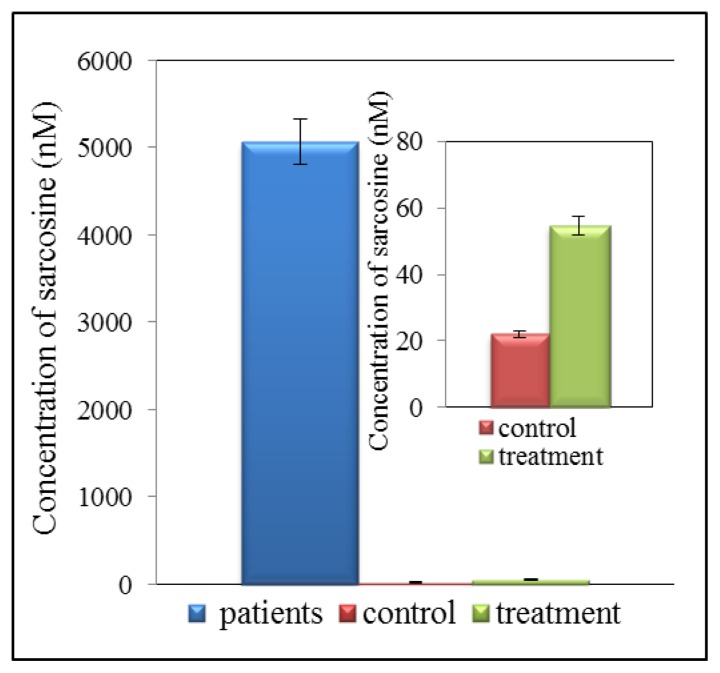
Overview of concentration of sarcosine in urine of patients suffering from prostate cancer (CaP), treated patients and controls (healthy people).

Detection of biomarkers using liquid chromatography (LC) is currently one of the most widely used analytical techniques. Advantage of LC application consists in repeatable and high throughput detection of low molecular mass compounds. It is therefore possible to detect and quantify very low concentrations of analytes in the presence of various interfering components. Very good results have been obtained by using chromatography in tandem with mass spectrometry used for determination of sarcosine in urine samples. This method is highly sensitive and allows determining sarcosine in very low concentrations. However, there is one great advantage, this method is able to separate sarcosine from its isomers and alanine, which may be responsible for a false-positive and false-negative results. Jentzmik *et al.* used gas chromatography with tandem mass spectrometry to determine sarcosine [7]. It was detected in the samples of both malignant and non-malignant tissues after radical prostatectomy from 92 patients suffering from prostate cancer. Significant differences in concentrations of sarcosine in malignant and non-malignant tissues were found. Concentration of sarcosine was more than 7% higher in malignant tissues compared to non-malignant. Based on these results it was concluded that sarcosine could not be considered as a suitable predictor of CaP [7]. In another published study gas chromatography (GC) and mass spectrometry (MS) were used. The results showed that the sarcosine/alanine ratios in patients with early and advanced prostate cancer were fairly constant showing no statistically significant differences between T-stages [61]. It was therefore concluded that sarcosine is not a suitable marker for prostate cancer. By comparing PSA with T-stages in the same group of patients it was found that PSA in the T1–T2 group of patients was significantly lower than in the T3–T4 group of patients confirming the well know merits and limitations of this marker [61]. Reverse phase high performance liquid chromatography (HPLC) is the most utilized technique for separation of amino acids [62,63] or peptides [64]. HPLC with tandem mass spectrometry, which is presently one of the most widely used analytical methods, was used to determine sarcosine and six other metabolites in urine [65]. This method is able to determine sarcosine in much lower levels than gas chromatography. The technique developed in this study is very simple, fast, sensitive, robust and repeatable. Cernei *et al.* showed that electrochemical detection is also a suitable method to detect sarcosine in very low concentration. However, this method can be used to analyse real samples only after application of suitable pre-treatment [21,66]. Application of ion-exchange liquid chromatography is also suitable to separate amino acids [21]. It can be used to analyse all organic compounds that contain amino acids [67]. Ion-exchange chromatography method developed by Cernei *et al.* revealed that level of sarcosine in urine of patients suffering from CaP is several times higher than that of cured patient (Figure 4). The level of sarcosine in healthy patients is only negligible [21]. Table 1 summarizes the method used for sarcosine detection.

## 7. Summary of Results Obtained by Analysis of Samples from CaP Patients

Table 2 shows different types of prostate cancer diagnosed and published in the studies. Sarcosine has been determined in majority of presented works. Despite the fact that some publications do not claim sarcosine to be a potential marker for CaP, sarcosine was detected and quantified in almost all studies in amounts different from the controls. This statement corresponds with results by Khan *et al.* They validated sarcosine as an important oncometabolite using both *in vivo* and *in vitro* preclinical models. Moreover they confirmed that overexpression of *GNMT* in cells elevated also the sarcosine levels but had no effects on cell proliferation [68].

Table 2 reveals the connection between sarcosine and prostate cancer. Sarcosine has been shown to be rapidly released into the urine supernatant [69]. Compared to study of Sreekumar *et al*. [1], Jentzmik *et al.* [69] suppose that the data by Sreekumar *et al.* [1] are more likely a result of cohort differences rather than true elevations of sarcosine levels in urine of patients suffering from prostate cancer [7]. Struys *et al.* [70] compared serum sarcosine concentrations between three groups of men, one of them was created by CaP patients, second were patients with increased PSA and the third one were individuals who have been assessed for vitamin B12 status. Results obtained by Struys and colleagues demonstrate that the mean of concentrations of sarcosine in serum does not discriminate between the three groups of men. The concentrations of sarcosine also did not demonstrate a correlation with tumour progression or PSA [70]. Despite this fact, other results shown in Table 2 more or less confirm the sarcosine as a potential CaP marker.

Moreover, Wu *et al.* [71] determined sarcosine in urine samples of patients suffering from CaP, and the authors finally conclude that value of sarcosine determined in urine has limited potential in the diagnostic algorithm of CaP. The authors suggested that the parameter sarcosine/creatinine ratio had not been accurate enough to diagnose CaP. In addition, it cannot reliably predict the histologic grade and behaviour of a tumour [71]. Lucarelli *et al.* [59] concluded that higher serum sarcosine levels were significantly associated with low- and intermediate-grade tumours in men with PSA < 4 ng/mL, nevertheless it is important to combine serum sarcosine with other tumour markers, such as PCA3 and [−2] pro PSA. Thereafter it could have an important role in selecting men with low grade/low risk PCa who should undergo active surveillance programs [59]. Bianchi *et al.* [72] concluded that sarcosine cannot be considered as a reliable marker for prostate cancer in urinary sediments. The analysis of a more extended number of samples taken from both healthy and CaP-diseased patients (localized and metastatic cancer) would be, according to extensiveness of issue, helpful to clarify definitively the role of sarcosine.

## 8. Conclusions

Non-proteinogenic amino acid sarcosine has a promising potential as a non-invasive marker of carcinoma of prostate as well as GNMT, which is closely connected with the presence and involvement of sarcosine in tissue of prostate tumours. Analytical demands on accurate and precise detection of low concentrations of sarcosine are high, and relatively expensive instrumentation is quite necessary. However, the limits of detection in units of nanograms can be achieved and reversed this fact. The finding that sarcosine does not occur in urine of healthy patients in detectable levels [1,5] and the necessity to eliminate negative results are very important for developing the low-cost, rapid, and reliable analytical methods.

## Figures and Tables

**Table 1:** Comparison of methods used for determination of sarcosine in different matrixes. In addition, the necessity to pre-treat samples is also indicated.

Method	Matrix	Sample pre-treatment	Ref.
Microarray-based analysis	DNA	Medium	[5]
GC/MS	Urine, tissue, serum	High	[61]
LC/MS	Urine, tissue, serum	High	[65]
LC/ED	Urine, tissue, serum	High	[21]
IEC	Urine, tissue, serum	High	[21]

GC/MS: gas chromatography with mass spectrometry; LC/MS: liquid chromatography with mass spectrometry; LC/ED: gas chromatography with electrochemical detection; IEC: ion exchange chromatography.

**Table 2:** Overview of the published papers containing data about changes in level of sarcosine in samples of urine of patients with diagnosed prostate cancer.

Number of patients	Diagnosis	Sarcosine	Ref.
10	Non-specific prostate cancer	+	[47]
29	Low differentiated acinar prostate adenocarcinoma	+	[21]
14	Metastatic prostate cancer	+	[1]
71	Medium differentiated prostate acinar adenocarcinoma	+	[21]
106	Non-specific prostate cancer	−	[69]
15	Non-specific prostate cancer	+	[71]
10	Acinar adenocarcinoma of prostate	+	[21]
33	Metastatic prostate cancer	+	[72]
86	Non-specific prostate cancer	+	[19]
13	Medium differentiated prostate acinar adenocarcinoma	+	[73]
3	Metastatic prostate cancer	+	[65]
18	Metastatic prostate cancer	+	[70]
290	Metastatic prostate cancer	+	[59]

+ means elevated sarcosine in CaP patients; − means decreased sarcosine in CaP patients.
